# Transgender patient undergoing Rezum therapy: a case report

**DOI:** 10.1093/jscr/rjae064

**Published:** 2024-02-13

**Authors:** Om V Sakhalkar, Luke Scanlan, Zachary Klaassen, Sherita A King, Matthew N Simmons, Martha K Terris, Pablo J SantaMaria

**Affiliations:** Medical College of Georgia at Augusta University, Augusta, Georgia, United States; Medical College of Georgia at Augusta University, Augusta, Georgia, United States; Medical College of Georgia at Augusta University, Augusta, Georgia, United States; Medical College of Georgia at Augusta University, Augusta, Georgia, United States; Medical College of Georgia at Augusta University, Augusta, Georgia, United States; Medical College of Georgia at Augusta University, Augusta, Georgia, United States; Medical College of Georgia at Augusta University, Augusta, Georgia, United States

**Keywords:** Rezūm, transgender, benign prostatic hyperplasia, safety

## Abstract

Rezūm is a relatively new, minimally invasive approach that utilizes vaporized water to ablate prostatic tissue surrounding the proximal urethra in patients with benign prostatic hyperplasia. However, of the many notable studies involving Rezūm’s effectiveness in men, none have documented use of Rezūm in the transgender community. With a growing population of transgender patients in the USA, prostate treatments will be offered for transgender women on a more regular basis. To the best of our knowledge, we introduce the first case of Rezūm being utilized to treat benign prostatic hyperplasia in a patient self-identifying as a woman.

## Introduction

**Figure 1:**
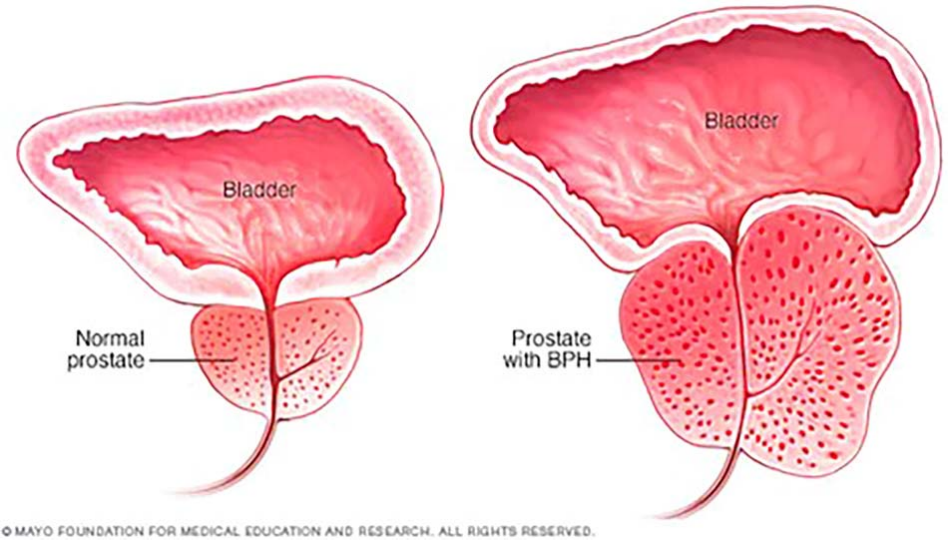
Depiction of BPH [3].

**Figure 2:**
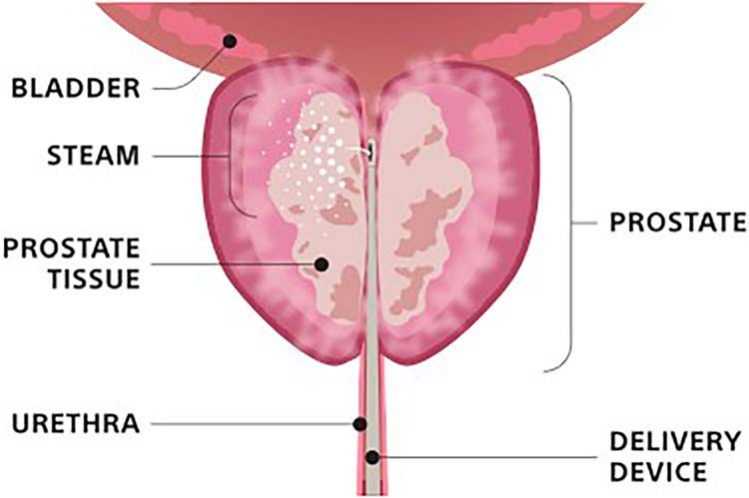
Demonstration of Rezūm Procedure [5].

**Figure 3:**
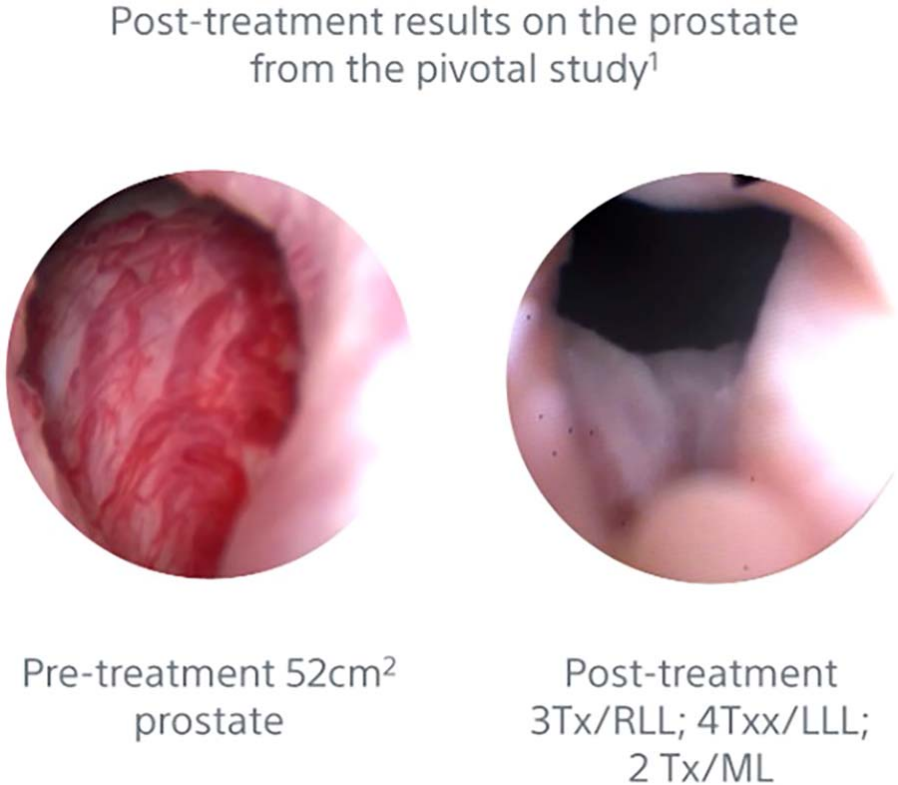
Demonstration of Rezūm procedure highlighting the pre- and post-treatment results [6].

Benign prostatic hyperplasia (BPH) continues to be an extremely common process affecting ~50% of men between the ages of 50 and 60 years old, with increasing rates as men age [1]. While many cases of BPH may have few obstructive or bothersome effects on patients, severe cases can lead to urinary retention, recurrent infections, and bladder stones [2]. Figure 1 demonstrates how BPH impacts these urinary symptoms [3]. Current treatment guidelines utilize alpha-1 reductase inhibitors as monotherapy or in combination with 5-alpha reductase inhibitors to improve lower urinary tract symptoms (LUTS) secondary to an enlarged prostate [4]. Once a patient has failed pharmacologic treatments, they may turn to surgical or procedural options. Figures 2 and 3 demonstrate how Rezūm is one such minimally invasive approach, which utilizes vaporized water to ablate prostatic tissue surrounding the proximal urethra [4–6]. Figure 3 provides a detailed description of Rezūm 6 months after the procedure in a different pivotal study, with three treatments to the right lower lobe, four treatments to the left lower lobe, and two treatments to the middle lobe [6]. Rezūm has been labeled as a safe, effective, and durable treatment option for men with moderate to severe LUTS [7]. However, of the many notable studies involving Rezūm’s effectiveness in men, none have documented use of Rezūm in the transgender community. Here, we introduce the first case of Rezūm being utilized to treat BPH in a patient self-identifying as a woman.

## Case report

We present a 68-year-old transgender female patient who has a past medical history of BPH, lichen sclerosis and vaginal irritation, hyperlipidemia, hypertension, post-traumatic stress disorder, sleep apnea, prediabetes, and coronary artery disease. Her past surgical history includes penile inversion with labiaplasty and orchiectomy 10–15 years ago as well as multiple stents for coronary artery disease. She used to wake up three to four times nightly to urinate, leading to her sleeping only 3–4 h per night. She also endorsed urgency during the day, leakage one to two times every week, and frequency. She self-reported an International Prostate Symptom Score (IPSS) of 17, indicating moderate symptoms. A preprocedural urodynamics study determined a Qmax (flow rate) of 16 ml/s and a postvoid residual of 120 ml. She has had no episodes of acute urinary retention. Before her Rezūm procedure, she was only Foley-dependent for 1 week following her facial feminization and breast augmentation surgical procedures and has had no episodes of high PVR. She had a past GU-related infectious history of *Candida vulvovaginitis* with two separate instances of *Escherichia Coli* presence in her urine within a 6-month period, with the second *E. Coli* infection being days prior to Rezūm. Her past medical and surgical history of coronary artery disease with stenting made her an ideal candidate for a minimally invasive procedure such as Rezūm. She had a preprocedural prostate volume of 35 cc with a prostate length of 2.5 cm. She received 100 mg gentamicin and 1000 mg ampicillin preoperatively. She underwent the Rezūm procedure. The patient was placed into the supine position and into stirrups. She was prepped and draped in our usual sterile fashion. The Rezūm device was placed per urethra under direct visualization with a 30° lens and saline irrigation. Each lateral lobe was treated two times. The median lobe was not present, and the central zone was not treated. The cystoscope was removed. A 16 French Foley catheter was placed per urethra with immediate return of clear yellow urine. The balloon was inflated with 10 cc of sterile water. She was discharged the same day without complications. She received no analgesics postoperatively except over-the-counter analgesia. Indwelling Foley catheter was removed 5 days postoperatively, and the patient reports doing well.

## Discussion

Minimally invasive surgical techniques (MISTs), such as Rezūm, have been expanded over the past few decades. Many of these MISTs provide numerous advantages in comparison to medical therapy and the conventional transurethral resection of the prostate (TURP) [8, 9]. This has driven the demand for MISTs. One advantage of MISTs such as Rezūm is the reduction in side effects compared to both pharmacologic therapy and TURP as well as improvement in overall adherence compared to pharmacologic therapy. MISTs also prove to be a potentially quicker solution to LUTS when compared with medical therapy and have a better side effect profile when compared to TURP. Overall, MISTs preserve sexual function, avoid long-term medication use, and have fewer side effects compared to both medical therapy and TURP. Nonetheless, MISTs are novel, and long-term efficacy and side effects remain debatable. The need for follow-up procedures after MISTs must be acknowledged, and further research is needed to better understand this potential disadvantage [8, 9].

Rezūm is a relatively new procedure which was granted FDA approval in 2015 [10]. Its indication is to treat LUTS caused by BPH and has proven to be a successful procedure. With an IPSS, quality of life, and Qmax improvement of around 50%, Rezūm will continue to improve the lives of large populations of patients [4]. However, as with most prostate targeting procedures, the population of patients undergoing Rezūm continues to be those identifying as men. To the best of our knowledge, this procedure has not yet been documented in literature as being performed on transgender women. With a growing population of transgender patients in the USA, prostate treatments will be offered for transgender women on a more regular basis [11]. As with any future patient, the commitment to excellence and transparency is of utmost importance. This case report provides reassurance to urologists that Rezūm has been successfully performed in transgender women and can be applied to similar patients accordingly. This case study also provides reassurance to transgender women who may have concerns about the efficacy and safety profile of the Rezūm procedure.
